# Relevance Proof of Safety Culture in Coal Mine Industry

**DOI:** 10.3390/ijerph16050835

**Published:** 2019-03-07

**Authors:** Wei Jiang, Chunyang Liang, Wei Han

**Affiliations:** 1 College of Emergency Management & Safety Engineering, China University of Mining & Technology(Beijing), Beijing 100083, China; liangcy05@126.com (C.L.); hanwei2324@126.com (W.H.); 2 School of Humanities, Languages and Social Science, Nathan campus, Griffith University,170 Kessels Road, Nathan, Brisbane, Queensland 4111, Australia

**Keywords:** safety culture, safety management, safety knowledge, safety awareness, safety habits

## Abstract

This paper intends to use data to verify the correlation between safety culture, safety management system and safety knowledge, safety awareness, and safety habits, which is the correlation between the various parts of the behavior safety “2-4” model. Due to data limitations, the results are limited to the study of safety culture related relationships in coal mining enterprises. This paper first designed a questionnaire containing 30 questions, of which 1–5 questions represent safety culture, 6–22 questions represent safety management system, and 23–30 questions represent safety knowledge, safety awareness and safety habits. Employees of 27 coal mining enterprises in Shandong, Henan, Hunan and other places in China were surveyed and sampled by stratified random sampling, and 1514 valid questionnaires were obtained. After item analysis and correlation analysis, and it was found that, within the data of 1514 questionnaires, the item total correlation coefficients of questions 6, 9, 19 and 28 were all less than 0.2, indicating that the identification degree of these four items was poor, which was deleted. Using the data analysis of the remaining 26 questions in the questionnaire, it was found that the relationship between safety culture and the safety management system, the safety management system and safety knowledge, and safety awareness and safety habits is moderately related; safety culture and safety knowledge, safety awareness and safety habits are weakly related. The conclusion shows that the safety culture directly affects the safety management system; the safety management system directly affects the safety knowledge, safety awareness and safety habits; the safety culture indirectly affects safety knowledge, safety awareness and safety habits. However, why the expected strong correlation is not achieved, and whether the same conclusion can be obtained if the data scale is expanded or other types of enterprises are added for questionnaire measurement, these are questions worthy of further study, which is also the author’s next research content.

## 1. Introduction

**Figure 1:**
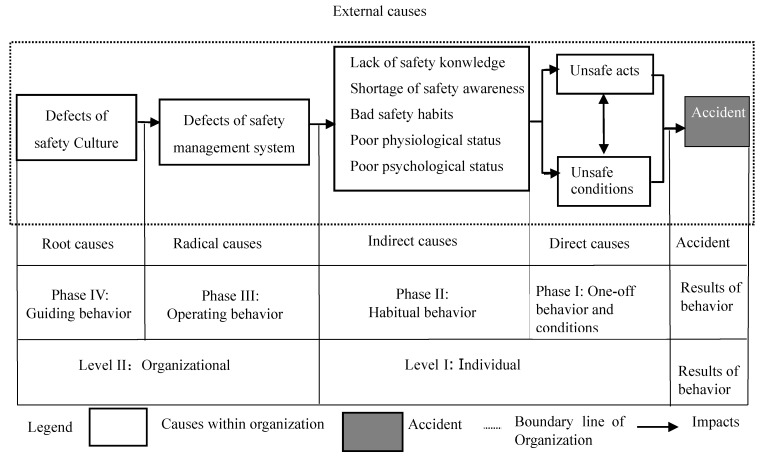
Accident causation “2-4” model (24 Model).

Professor Fu in the project team where the author works put forward the behavior safety “2-4” model [1], as shown in Figure 1 below. The project team members used the model to analyze 748 coal mine accidents. For details, see the website information in Ref. [2] and several published papers. The paper deals with coal mine accident analysis [3] and accidents in other industries [1,4,5,6,7,8]. The behavior safety “2-4” model points out that safety culture directly affects the safety management system, the safety management system directly affects people’s safety knowledge, safety awareness, safety habits, safety physiological and safety psychology, and safety culture indirectly affects safety knowledge, safety awareness, safety habits, etc. through the safety management system. However, the correlation between the safety culture, safety management system, safety knowledge, safety awareness and safety habits that caused the accident has not been studied. Therefore, this paper intends to use the questionnaire design method to obtain data to verify the correlation between safety culture, safety management system and safety knowledge, safety awareness and safety habits.

Ref. [9] analyzes the research status of China’s safety culture, and introduces the development, achievements and prospects of China’s safety culture, in which the author and the member of the project team (Gui Fu, Wei Jiang) are mentioned. This article is also a continuation of the safety culture research content of the author and author’s research team.

At present, scholars have studied the relationship between safety culture and safety management, safety culture and safety performance, safety culture and other related content. These studies are both qualitative and quantitative. 

Regarding the relationship between safety culture and safety management, many scholars have found that safety culture has a direct or indirect impact on safety management. For example, Guldenmund [10] analyzed the safety culture of the core, performance and overall dimensions. It is concluded that, through the combination of these three safety cultures, the concept of safety culture can be reshaped and the analytical potential of safety culture in understanding the development and implementation of safety management systems can be enhanced. Robertson [11] conducted a safety culture survey using the university aviation program and found a relationship between safety management implementation and safety culture, safety improvement and safety culture, management commitment and safety culture. Hendershot [12] found that an effective process safety culture is very important to achieve effective process safety management, and a good safety culture is the key to excellent process safety management. Lin et al. [13] found that the intensity of safety culture has a direct or indirect impact on the safety management system and can affect the safety performance of the company. Rollenhagen and Wahlström’s [14] research shows that the safety of nuclear power plants cannot always be based on prescribed instructions. The management system and safety culture should solve this problem, and should better understand the structure and content of the management system and their impact on the safety of nuclear power plants. McDonald et al. [15] studied various aspects of the safety management system and safety culture of the four organizations and found that the safety management systems and safety culture of the four organizations are largely consistent. There is no difference between the four organizations’ adherence to mission procedures and safety attitudes, but with different subcultures, indicating differences in the concept of safety culture. Grote [16] studied the uncertainty in management and proposed that safety culture is one of the means. It is suggested that the different roles and importance of safety culture depend on the selected uncertainty management method. It is required that any safety culture assessment must be evaluated through the matching between the uncertainty faced by the organization and the uncertainty forms selected to deal with. Gao et al. [17] established a model to explore the mediating role of safety management practices in the development of process safety culture. Four safety management practices, including organizing responsibilities/procedures, communication and coordination, safety training, and inspection and monitoring, have been found to have positive mediating effects on safety culture, with inspection and monitoring presenting the highest mediating effect.

For the research on safety culture and behavior, Fung et al. [18] investigated the relationship between people’s safety culture and behavior, attitude and cognition, and made a comparison. The research showed that organizational commitment has a high correlation with communication, supervisors, individuals, workers, risk-taking behavior and other aspects. Tylor [19] studied the safety culture model associated with the organization’s shared safety awareness to observable safety behavior, and used accident cases to study the relationship between safety culture and observable safety behavior. Ismail et al. [20] evaluated the impact of environmental behavior factors on the development of safety culture and found that the internal environment of the organization is weak and will affect safety behavior. In addition, through interviews with senior management, it was discovered that the company’s safety culture status was contributed by the established system. The Nævestada et al. [21] study found that safety cultures at different analytical levels influence different types of unsafe behaviours, which in turn influence the risk of work injuries. It is recommended to study the impact of culture on traffic safety at different levels of analysis (i.e., countries, departments, and organizations).

Regarding safety culture and safety performance research, Kalteh et al. [22] uses the keywords “Safety Culture and Safety Performance” and “Safety and Performance” to select English articles published in 2005–2017 from different databases, and evaluates the role of safety culture in safety performance in 31 articles extracted. It is found that improving the safety culture level can effectively reduce accidents and improve safety performance indicators. Feng et al. [23] uses a variety of techniques to collect data from 47 completed construction projects in Singapore. Research shows that the safety performance of construction projects depends on the synergies between safety investment, safety culture and engineering hazards. Chen et al. [24] explored the interactive relationship between Taiwan’s construction industry safety culture and corporate safety performance, and found that safety culture has certain predictive power and influence on Taiwan’s construction industry safety performance.

There are also related studies on the relationship between safety culture or safety management system and knowledge. For example, Vinodkumar et al. [25] used a questionnaire survey to measure employees’ cognition of six safety management practices and self-reported safety knowledge, safety motivation, safety compliance and safety participation. It turns out that safety knowledge and safety motivation are key factors in interpreting these relationships. Firoozi et al. [26] found that knowledge is an important source of capital for an organization and can only be correctly created and managed in a healthy cultural environment. The paper adopts a health, safety, environment and cultural model as a management model, which enables people to have a deep understanding of the health and safety culture that promotes the interaction and transformation process. Azer et al. [27] explored the relationship between organizational culture, knowledge management, and patient safety performance. The study found that different dimensions of organizational culture are associated with more effective knowledge management, and knowledge management is associated with better patient safety performance. Warszawska et al. [28] identified six aspects of safety culture through safety culture assessments: knowledge and skills, awareness, information flow, monitoring and supervision, management commitment, and continuous improvement. Kuimet et al. [29] studied the links and developments between human resource management, organizational safety culture and knowledge management literature, which found that the concept and framework of human resource management can play an important role in the communication of safety knowledge within the organization.

This paper intends to use data to verify the relevance between safety culture, safety management system and safety knowledge, safety awareness, and safety habits. 

## 2. Description of Research Methods and Content

**Figure 2:**
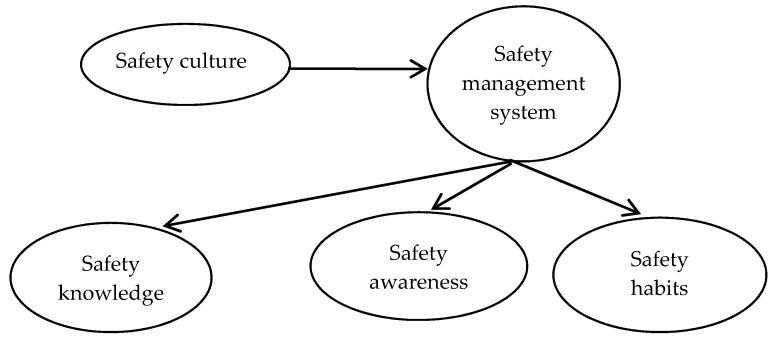
Correlation between safety culture, safety management and safety knowledge, safety awareness, safety habits.

This paper intends to use the questionnaire design method to obtain data to verify the correlation between safety culture, safety management system and safety knowledge, safety awareness and safety habits, as shown in Figure 2 below.

The research method is to analyze the contents of safety culture, safety management, safety knowledge, safety awareness and safety habits in the literature [1], design multiple questions for each part of the content, form a questionnaire, and use the SPSS software (SPSS Inc., Chicago, IL, USA) to analyze the data obtained from the questionnaire. Finally, the correlation results of safety culture, safety management, safety knowledge, safety awareness and safety habits are obtained.

The definitions and contents of safety knowledge, safety awareness, safety habits, safety management system and safety culture are shown in literature [30]. It should be explained that this article does not attempt to separate safety knowledge, safety awareness and safety habits, but to use them as a concept to verify its relevance to safety culture and safety management. The reason is that, in the behavior safety “2-4” model, insufficient safety knowledge, poor safety awareness and poor safety habits are all indirect causes of accidents.

## 3. The Questionnaire Design

For the meaning of each part of safety culture, safety management system, safety knowledge, safety awareness, and safety habits, set up multiple questions, form a questionnaire, and outline the content of each question, as shown in Table 1 below.

## 4. The Selection and Basic Situation of the Investigated Personnel

Since the distribution, filling and collection of the questionnaire will affect the daily work of the enterprise, and the questionnaire needs to be filled out voluntarily by the enterprise. After the inquiry, 27 coal mine enterprises are willing to assist in filling out the questionnaire. These 27 coal mine enterprises are located in Shandong, Henan, Hunan and other places in China. In each enterprise, stratified random sampling was used to sample a total of 1610 employees were selected, and 1610 questionnaires were issued. Excluding the questionnaires with too much reaction tendency and missed questions, 1514 valid questionnaires were obtained, and the effective recovery rate was 94%.

This section counts three parameters of the basic situation of the tested personnel: personnel structure, education level and working years.

### 4.1. Personnel Structure

**Figure 3:**
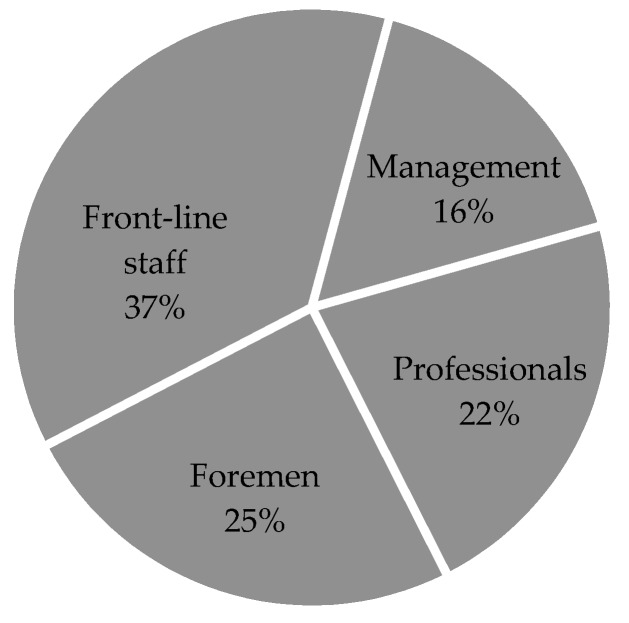
Personnel composition ratio diagram.

The investigated personnel were mainly divided into four categories: (1) Management, that is, cadres at the middle level or above; (2) Foreman, that is, the leader who directly leads the front-line staff or “leads the team”, usually the squad leader, the team leader, etc.; (3) professional, called non-leading cadres in China, are characterized by higher consciousness and higher education level compared with front-line staff, and can generally complete a certain task independently; and (4) front-line staff, that is, those who directly perform on-site operations. The stratified random sampling method was used to extract testers. According to the proportion of each type of personnel, 249 management personnel, 332 professionals, 376 foremen, and 557 front-line staff, totaling 1,514 people, were selected, as shown in Figure 3.

### 4.2. Educational Level

**Figure 4:**
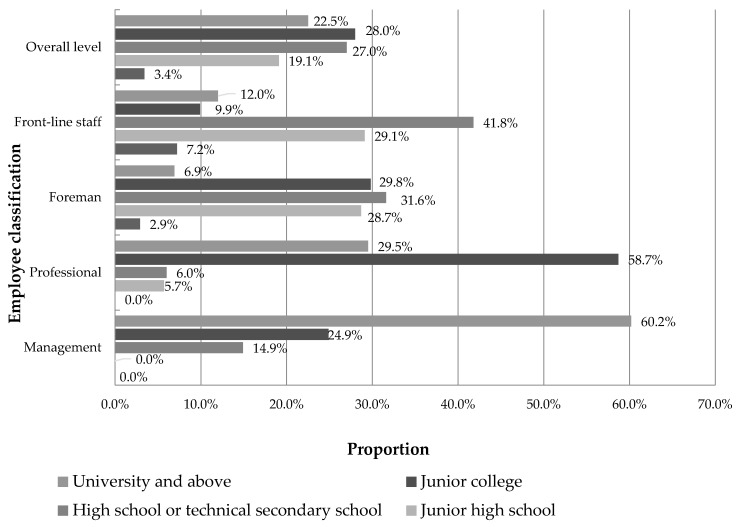
Basic information on the educational level of the tested employees.

Figure 4 describes the degree of education of employees in the coal mine enterprise under investigation. Among the “overall personnel”, high school education and the above account for a relatively high proportion, reaching 77.5%, indicating that the overall level of cultural quality of the tested personnel is relatively high. Among them, the number with junior college education is the largest, accounting for 424 people, and accounting for 28% of the total sample. The lowest number of people with an education level below junior high school was 51, accounting for 3.4% of the total sample. The education level of the management and professionals is mostly junior college or above, accounting for 85.1% and 88.2%, indicating that the cultural quality of the management and technical personnel in the tested coal mines is relatively high.

### 4.3. Working Years

**Figure 5:**
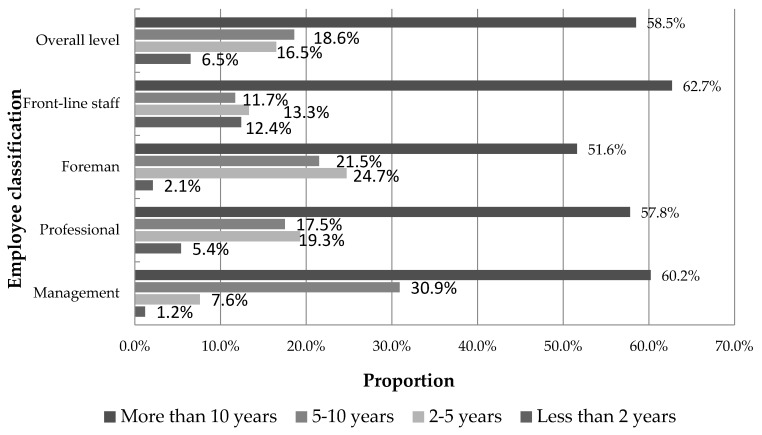
Basic situation of the working years of the tested employees.

Figure 5 describes the working years of coal mine employees in the coal mine enterprise under investigation. The proportion of employees with “10 years of work experience” in “management, foremen, professionals, front-line staff” was 60.2%, 57.8%, 51.6%, and 62.7%, respectively; the proportion of “all personnel” was 58.5%. These people have accumulated a lot of technical and management experience in the long-term work. In work practice and training, these employees can tell more about their personal experience and lessons, which is helpful for enterprise safety work and other related work, and can reduce the accident rate of enterprise safety production.

## 5. Questionnaire Measurement Results Item Analysis

The item analysis mainly analyzes the discrimination degree of the questionnaire items, and identifies whether the questionnaire items can clearly distinguish the degree of reaction of different test subjects. Item analysis is conducted for each safety culture level questionnaire item, and those items that are not discriminating or not highly discriminating are the items of the measurement questionnaire that need to be deleted.

### 5.1. Critical Ratio Method

The item analysis of the questionnaire uses the critical ratio method. Firstly, reverse scoring was performed on the reverse questions in the measurement items, and the total scores of all tested personnel on the measurement scale were calculated; the upper and lower 27% are the grouping criteria, and the first 27% of the total measurement score is set as high grouping, and the last 27% of the total measurement score is set as low grouping; Using the Independent sample *t*-test, the significant difference between the average scores of the high and low groups on each measurement item is tested. If the result of the *t*-test reaches a significant level, it indicates that the measurement item can identify the response of different test subjects, that is, the item has a high degree of discrimination. If the result of the *t*-test does not reach the significance level, or the *t*-statistic of the difference between the high and low group of the item is less than 3, it means that the degree of discrimination of the item is poor, and it may be considered to delete it. In this paper, reverse scoring is carried out for the reverse items in the measurement items, and the total score of all items is calculated, and the ranking is conducted according to the score. SPSS software was used to conduct the Independent sample *t*-test, and the mean significance difference between the low group and the high group on 32 items was obtained. The test results are shown in Table A1.

From the results of the Independent sample *t*-test in Table A1, the *t*-statistic values of the differences between high and low groups of the 30 items in the questionnaire are all greater than 3, and the significance level of the *t*-test is 0.000, indicating that the 30-measurement item has a high degree of discrimination and can identify the responses of different test groups.

### 5.2. Correlation Method

The correlation method is to calculate the correlation between the scores of each item and the total score of all items, that is, the total correlation coefficient of the items. The items with a total correlation coefficient less than 0.20 should be eliminated; the item with a total correlation coefficient between 0.20 and 0.29 can be barely used; the items with a total correlation coefficient between 0.30 and 0.39 are good and will be retained; the items with a total correlation coefficient greater than 0.40 are excellent and will be retained [31]. This paper calculates the correlation coefficient between the scores of 30 questions in the questionnaire and the total score. The results are shown in Table 2.

It can be seen from Table 2 that the total correlation coefficient of the item in questions 6, 9, 19 and 28 is less than 0.2, indicating that the discrimination of these four items is poor and will be deleted. The total correlation coefficient of other items is between 0.20 and 0.50, and the item identification is better. The data of the remaining 26 questions are used to prove the relationship between safety culture, safety management system, safety knowledge, safety awareness and safety habits.

## 6. Correlation Verification between Safety Culture and Safety Management System and Safety Knowledge, Safety Awareness and Safety Habits

By analyzing the data, the sample distribution is not particularly consistent with the normal distribution, and there is no linear relationship between the two. Therefore, Spearman rank correlation coefficient in correlation analysis is selected for analysis, and the value range of the correlation coefficient in the analysis result is between −1 and +1. The larger the absolute value is, the stronger the correlation was, and the symbol indicates the correlation direction. In addition, 0–0.09 is not correlated, 0.1–0.3 is weakly correlated, 0.3–0.5 is moderately correlated, and 0.5–1.0 is strongly correlated [32,33]. Table 3, Table 4 and Table 5 list the correlation coefficients between the three categories.

This paper studies the correlation between safety culture, safety management and safety knowledge, safety awareness, and safety habits. Since the behavior safety “2-4” model believes that safety culture directly affects safety management, and safety management directly affects safety knowledge, safety awareness, and safety habits, it is expected that safety culture and safety management are related, preferably having a strong correlation. The same is the relationship between safety management system and safety knowledge, safety awareness, and safety habits. However, the results obtained at the end of this paper are found to be moderately related, indicating that the relevant relationship to be proved in this paper is successful, but further research is needed to prove whether the correlation can achieve strong correlation and what factors affect the correlation. This is the content of the next study. In the behavior safety “2-4” model, the safety culture indirectly affects safety knowledge, safety awareness, and safety habits through safety management system. The expectation is to prove that safety culture is related to safety knowledge, safety awareness and safety habits. The results obtained in this paper prove that the two are correlated and weakly correlated. 

Therefore, this paper has succeeded in determining the correlation between safety culture, safety management and safety knowledge, safety awareness and safety habits through the questionnaire survey. The next step is to further study whether the relationship between safety culture and safety management, safety culture and safety knowledge, safety awareness, and safety habits can achieve strong correlation and factors affecting the correlation.

## 7. Conclusions

In summary, the following conclusions are obtained:

(1) This paper designed a questionnaire with 30 questions, among which 1–5 questions represent safety culture, 6–22 questions represent safety management system, and 23–30 questions represent safety knowledge, safety awareness and safety habits.
(2) The questionnaires designed in this paper were used to measure the employees of 27 coal mines in Shandong, Henan, Hunan and other places in China. Sampling was carried out by stratified random sampling, and 1514 valid questionnaires were obtained.
(3) After project analysis and correlation analysis, it was found that the total correlation coefficients of questions 6, 9, 19 and 28 were all less than 0.2, indicating that the identification degree of these four items was poor, which was deleted.
(4) Using the data analysis of the remaining 26 questions in the questionnaire, it was found that the relationship between safety culture and safety management system, safety management system and safety knowledge, safety awareness, and safety habits are moderately related. Safety culture is weakly related to safety knowledge, safety awareness and safety habits through the analysis of the Spearman rank correlation coefficient between safety culture and safety management system and safety knowledge, safety awareness and safety habits. The correlation coefficient between the safety culture and safety management system is 0.429. The correlation coefficient between safety management system and safety knowledge, safety awareness and safety habits is 0.376. The correlation coefficient between safety culture and safety knowledge, safety awareness, and safety habits is 0.291.
(5) The conclusions in this paper can only prove the correlation and cannot prove the causal relationship.

## Figures and Tables

**Table 1:** Overview of questionnaire setting contents.

Category	Questionnaire Subject Number	Summarization of Questionnaire Subject Content
Safety culture	1	The importance of safety
	2	The degree to which casualties can be prevented
	3	Safety creates economic benefits
	4	The degree of safety integration into corporate management
	5	Safety is mainly determined by safety awareness
Safety management system	6	Awareness of safety inputs
	7	The role of safety regulations
	8	Degree of responsibility of the management
	9	The role of the safety department
	10	Degree of employee involvement
	11	The role of the linear department
	12	The role of the management system
	13	Quality of safety meetings
	14	The way of safety system formed
	15	The way of safety system implemented
	16	The type of accident investigation
	17	The type of safety inspection
	18	Caring for injured workers
	19	The degree of facility satisfaction
	20	The relationship between safety performance and human resources
	21	Safety management of subsidiaries and contract units
	22	Safety department’s work
Safety knowledge, safety awareness and safety habits	23	The subject responsibility of safety
	24	Formation of safety values
	25	Requirements for safety training
	26	Impact of community safety
	27	Amateur safety management
	28	The role of safety organization
	29	Overall safety expectation
	30	Emergency capability

**Table 2:** Total correlation coefficients of 30 questions.

**Question**	**1**	**2**	**3**	**4**	**5**	**6**	**7**	**8**	**9**	**10**
Correlation coefficient	0.338	0.451	0.364	0.309	0.327	0.196	0.446	0.326	0.185	0.470
**Question**	**11**	**12**	**13**	**14**	**15**	**16**	**17**	**18**	**19**	**20**
Correlation coefficient	0.285	0.338	0.363	0.378	0.404	0.326	0.383	0.269	0.153	0.367
**Question**	**21**	**22**	**23**	**24**	**25**	**26**	**27**	**28**	**29**	**30**
Correlation coefficient	0.322	0.452	0.336	0.330	0.329	0.289	0.261	0.097	0.300	0.350

**Table 3:** Correlation coefficient between safety culture and safety management system.

Control Variable	Coefficient	Safety Culture	Safety Management System
Safety culture	Correlation coefficient	1.000	0.429 **
	Significant (two-tailed)		0.000
	*N*	1514	1514
Safety management system	Correlation coefficient	0.429 **	1.000
	Significant (two-tailed)	0.000	
	*N*	1514	1514

** When the confidence level (double test) is 0.01, the correlation is significant.

**Table 4:** Correlation coefficient between safety management system and safety knowledge, safety awareness and safety habits.

Control Variable	Coefficient	Safety Management System	Safety Knowledge, Safety Awareness, Safety Habits
Safety management system	Correlation coefficient	1.000	0.376 **
	Significant (two-tailed)		0.000
	*N*	1514	1514
Safety knowledge, safety awareness, safety habits	Correlation coefficient	0.376 **	1.000
	Significant (two-tailed)	0.000	
	*N*	1514	1514

** When the confidence level (double test) is 0.01, the correlation is significant.

**Table 5:** Correlation coefficient between safety culture and safety knowledge, safety awareness and safety habits.

Control Variable	Coefficient	Safety Culture	Safety Knowledge, Safety Awareness, Safety Habits
Safety culture	Correlation coefficient	1.000	0.291 **
	Significant (two-tailed)		0.000
	*N*	1514	1514
Safety knowledge, safety awareness, safety habits	Correlation coefficient	0.291 **	1.000
	Significant (two-tailed)	0.000	
	*N*	1514	1514

** When the confidence level (double test) is 0.01, the correlation is significant.

## Appendix

**Table A1:** Independent sample *t*-test results.

Item Number		*t*-test for the Mean Value Equation						
		*t*	*df*	Sig. (Bilateral)	Mean Difference	Standard Error Value	95% Confidence Interval for Difference	
							Lower Limit	Upper Limit
1	Assume that the variances are equal	14.397	883	0.000	1.23184	0.08556	1.06391	1.39977
	Assume that the variances are not equal	14.406	690.665	0.000	1.23184	0.08551	1.06395	1.39973
2	Assume that the variances are equal	17.891	883	0.000	1.10298	0.06165	0.98199	1.22398
	Assume that the variances are not equal	17.900	755.908	0.000	1.10298	0.06162	0.98202	1.22395
3	Assume that the variances are equal	12.843	883	0.000	0.69252	0.05392	0.58669	0.79835
	Assume that the variances are not equal	12.850	735.533	0.000	0.69252	0.05389	0.58672	0.79833
4	Assume that the variances are equal	8.841	883	0.000	0.36566	0.04136	0.28448	0.44683
	Assume that the variances are not equal	8.850	458.004	0.000	0.36566	0.04132	0.28447	0.44685
5	Assume that the variances are equal	11.452	883	0.000	0.71957	0.06284	0.59624	0.84289
	Assume that the variances are not equal	11.459	659.967	0.000	0.71957	0.06279	0.59627	0.84287
6	Assume that the variances are equal	6.692	883	0.000	0.60645	0.09063	0.42857	0.78432
	Assume that the variances are not equal	6.692	876.725	0.000	0.60645	0.09062	0.42859	0.78430
7	Assume that the variances are equal	16.498	883	0.000	1.20135	0.07282	1.05843	1.34426
	Assume that the variances are not equal	16.507	717.692	0.000	1.20135	0.07278	1.05847	1.34423
8	Assume that the variances are equal	12.618	883	0.000	0.87601	0.06942	0.73975	1.01227
	Assume that the variances are not equal	12.619	879.059	0.000	0.87601	0.06942	0.73976	1.01226
9	Assume that the variances are equal	6.006	883	0.000	0.45980	0.07656	0.30954	0.61005
	Assume that the variances are not equal	6.006	877.323	0.000	0.45980	0.07655	0.30955	0.61004
10	Assume that the variances are equal	17.902	883	0.000	0.76694	0.04284	0.68286	0.85102
	Assume that the variances are not equal	17.912	707.911	0.000	0.76694	0.04282	0.68288	0.85101
11	Assume that the variances are equal	9.637	883	0.000	0.82060	0.08515	0.65348	0.98772
	Assume that the variances are not equal	9.640	826.186	0.000	0.82060	0.08512	0.65351	0.98768
12	Assume that the variances are equal	10.931	883	0.000	0.65516	0.05994	0.53753	0.77280
	Assume that the variances are not equal	10.937	724.975	0.000	0.65516	0.05990	0.53756	0.77277
13	Assume that the variances are equal	11.765	883	0.000	0.60212	0.05118	0.50168	0.70256
	Assume that the variances are not equal	11.774	630.005	0.000	0.60212	0.05114	0.50169	0.70255
14	Assume that the variances are equal	11.891	883	0.000	0.60700	0.05105	0.50682	0.70719
	Assume that the variances are not equal	11.901	564.523	0.000	0.60700	0.05100	0.50682	0.70718
15	Assume that the variances are equal	15.119	883	0.000	1.14937	0.07602	1.00016	1.29857
	Assume that the variances are not equal	15.119	882.985	0.000	1.14937	0.07602	1.00016	1.29857
16	Assume that the variances are equal	9.999	883	0.000	0.54617	0.05462	0.43897	0.65337
	Assume that the variances are not equal	10.009	514.020	0.000	0.54617	0.05457	0.43896	0.65337
17	Assume that the variances are equal	15.919	883	0.000	1.66174	0.10439	1.45686	1.86661
	Assume that the variances are not equal	15.920	877.448	0.000	1.66174	0.10438	1.45688	1.86660
18	Assume that the variances are equal	9.476	883	0.000	0.47577	0.05021	0.37724	0.57431
	Assume that the variances are not equal	9.482	712.333	0.000	0.47577	0.05018	0.37726	0.57429
19	Assume that the variances are equal	5.031	883	0.000	0.26946	0.05356	0.16433	0.37458
	Assume that the variances are not equal	5.032	855.296	0.000	0.26946	0.05355	0.16434	0.37457
20	Assume that the variances are equal	12.592	883	0.000	0.94071	0.07471	0.79409	1.08733
	Assume that the variances are not equal	12.600	667.577	0.000	0.94071	0.07466	0.79412	1.08731
21	Assume that the variances are equal	12.440	883	0.000	1.06256	0.08541	0.89492	1.23019
	Assume that the variances are not equal	12.447	704.971	0.000	1.06256	0.08537	0.89496	1.23016
22	Assume that the variances are equal	14.579	883	0.000	0.84860	0.05821	0.73436	0.96284
	Assume that the variances are not equal	14.592	530.931	0.000	0.84860	0.05815	0.73436	0.96284
23	Assume that the variances are equal	11.275	883	0.000	0.94396	0.08372	0.77965	1.10828
	Assume that the variances are not equal	11.277	874.349	0.000	0.94396	0.08371	0.77967	1.10826
24	Assume that the variances are equal	12.098	883	0.000	0.95245	0.07873	0.79793	1.10697
	Assume that the variances are not equal	12.098	882.987	0.000	0.95245	0.07873	0.79793	1.10697
25	Assume that the variances are equal	9.786	883	0.000	0.50081	0.05118	0.40036	0.60125
	Assume that the variances are not equal	9.794	553.709	0.000	0.50081	0.05113	0.40037	0.60124
26	Assume that the variances are equal	10.117	883	0.000	0.70531	0.06972	0.56847	0.84214
	Assume that the variances are not equal	10.119	853.795	0.000	0.70531	0.06970	0.56850	0.84211
27	Assume that the variances are equal	9.905	883	0.000	0.72459	0.07315	0.58102	0.86817
	Assume that the variances are not equal	9.905	882.608	0.000	0.72459	0.07316	0.58102	0.86817
28	Assume that the variances are equal	3.624	883	0.000	0.34728	0.09584	0.15919	0.53537
	Assume that the variances are not equal	3.624	877.259	0.000	0.34728	0.09583	0.15921	0.53536
29	Assume that the variances are equal	10.923	883	0.000	1.01634	0.09305	0.83372	1.19896
	Assume that the variances are not equal	10.920	849.614	0.000	1.01634	0.09307	0.83367	1.19901
30	Assume that the variances are equal	13.365	883	0.000	1.29584	0.09696	1.10555	1.48614
	Assume that the variances are not equal	13.367	872.502	0.000	1.29584	0.09695	1.10557	1.48612
